# Reactive arthritis of the temporomandibular joints and cervical spine in a child

**DOI:** 10.1186/1546-0096-5-4

**Published:** 2007-04-04

**Authors:** Bita Arabshahi, Kevin M Baskin, Randy Q Cron

**Affiliations:** 1 Division of Rheumatology, Department of Pediatrics, Children's Hospital of Philadelphia, PA, USA; 2 Division of Interventional Radiology, Department of Radiology, Children's Hospital of Philadelphia, PA, USA; 3 University of Pennsylvania School of Medicine, Philadelphia, PA, USA

## Abstract

**Background:**

Temporomandibular joint (TMJ) arthritis is frequently seen in children with chronic arthritis. It has rarely been described in a non-infectious acute setting. We report a case of reactive arthritis isolated to the TMJs and cervical spine.

**Case presentation:**

A 6-year-old Native American boy hospitalized for treatment of lymphadenitis and aseptic meningitis had an incidental brain magnetic resonance imaging (MRI) finding of effusions in the TMJs, as well as the atlanto-occipital and C1–C2 articulations. Repeat TMJ and cervical spine MRI four weeks later showed resolution of effusions. Reactive TMJ arthritis has been previously reported in adults but not in children.

**Conclusion:**

This report represents the first pediatric case of reactive arthritis isolated to the cervical spine and TMJs. Arthritis of the TMJ should be considered in the differential diagnosis of children with reactive arthritides.

## Background

A 6-year-old previously healthy Native American boy presented to the Emergency Department with a one-week history of right-sided anterior neck pain, progressing to swelling and fever and unresponsive to amoxicillin. Rapid group A Streptococcal antigen testing prior to initiation of amoxicillin was negative. He was admitted to the hospital with a diagnosis of lymphadenitis and started on intravenous (IV) clindamycin. He continued to be febrile up to 40.6°C despite antibiotic therapy, and received ibuprofen and acetominophen for management of fever. His physical examination was notable for trismus, right-sided neck swelling and erythema, and anterior right-sided neck pain limiting neck extension, but not flexion. On the third hospital day he developed a frontal headache and became increasingly somnolent and difficult to arouse. Head Computed Tomography (CT) was normal. Lumbar puncture revealed an elevated cerebrospinal fluid (CSF) protein of 80 mg/dL, a normal glucose level, red blood cells of 3 per cubic millimeter (mm^3^), and elevated white blood cells of 29 per mm^3 ^(21% neutrophils, 52% lymphocytes, 27% monocytes). His CSF and blood cultures were negative and he had normal Ebstein-Barr virus and Bartonella antibody titers. He was started on IV cefotaxime for broader antibiotic coverage but was presumed to have aseptic meningitis. His fever and neck swelling improved, but he continued to be somnolent.

**Figure 1:**
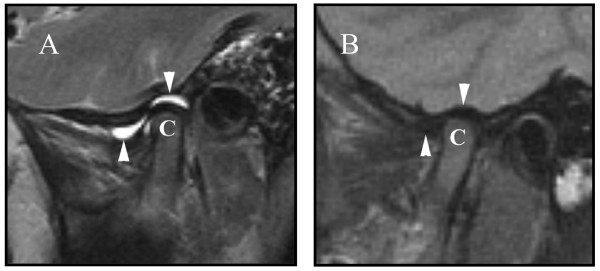
Image A is a T2-weighted proton density parasagittal MR image through the right TMJ showing effusions (arrows) just anterior to the condyle (C) in the superior and inferior synovial spaces. Follow up T2 MR image 4 weeks later (image B) is normal.

**Figure 2:**
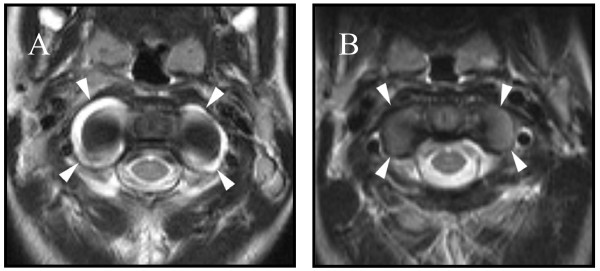
Image A is a T2-weighted proton density axial MR image through the C1–C2 articulations showing effusions (arrows) surrounding the joint spaces. Follow up T2 MR image 4 weeks later (image B) is normal.

A brain Magnetic Resonance Imaging (MRI) to evaluate his mental status revealed normal brain parenchyma, but marked presence of joint effusions in the temporomandibular joints (TMJ) (Figure 1A), the atlanto-occipital articulation, and the C1–C2 articulations (Figure 2A). He had not complained of posterior neck or TMJ pain, but his parents had noted on the day prior to the MRI, approximately 2 weeks after onset of his symptoms, he seemed to chew his food using a side-to-side, rather than up-and-down jaw motion. His mental status returned to normal the day after the MRI, and he had normal jaw translation, inter-incisor distance, and neck mobility with no associated pain. There were no effusions in any of his peripheral joints, and no note was made of pre-auricular swelling or erythema throughout his hospitalization. He had no history of recent otitis media, mastoiditis, or injury to his neck or jaw. Repeat TMJ (Figure 1B) and cervical spine (Figure 2B) MRI 4 weeks later revealed complete resolution of effusions, and his joint examination remained normal.

## Discussion

MRI evidence of effusions in the cervical spine and TMJs has been described in the pediatric population as a consequence of juvenile onset ankylosing spondylitis, juvenile rheumatoid arthritis, and trauma [1-3]. Other conditions such as septic arthritis, ganglion cysts, and synovial chondromatosis have also caused TMJ effusions, although their occurrence is generally associated with other clinical or radiographic signs suggestive of infection or neoplasm [4]. We describe a child with lymphadenitis and aseptic meningitis who developed transient TMJ and cervical spine effusions in absence of diagnostic exam findings.

MRI is gaining increasing popularity for the detection of arthritis because it can detect early inflammatory changes such as synovial proliferation and joint effusions preceding the development of cartilage destruction and bony erosions [2]. The presence of an effusion, in particular, signifies inflammation most commonly arising from trauma, infection, or chronic arthritis.

Septic arthritis of the TMJ most commonly occurs from hematogenous spread of organisms from a site of a distant infection. Infection may also spread from adjacent soft tissues, as is the case of TMJ septic arthritis following mastoiditis, middle or outer ear infections, and blunt trauma [5-7]. The most common organism involved is *Staphylococcus aureus*, and aspiration of joint fluid for culture confirms the diagnosis in 60% of cases [7].

By comparison, cervical spine septic arthritis is much more rare and often follows adjacent bone or soft tissue infection such as osteomyelitis, discitis, epidural abscess, and paraspinal abscess [4,8]. For the case reported herein, symptoms of trismus, abnormal chewing function, and decreased neck extension all subsided within 48 hours of broadening the antibiotic coverage. However, bilateral TMJ septic arthritis, particularly in the absence of facial swelling, would be highly unlikely. Furthermore, cervical spine septic arthritis, in the absence of adjacent bony derangements or abscess formation, has not been described.

Juvenile Rheumatoid Arthritis (JRA) and childhood spondyloarthropathies are frequently associated with arthritis of the cervical spine and TMJs; however, usually C2–C3 articulations are involved. Among the patients falling in this group, 17–87% are reported to have TMJ arthritis, and 40–62% develop cervical spine arthritis, particularly in late stages of disease [9,10]. Of note, TMJ arthritis in children is generally a painless process and not associated with any swelling detectable on examination [9]. However, TMJ or cervical spine arthritis as a sole presenting feature of JRA or spondyloarthropathy is rare. There are only two case reports, one of a teenager with TMJ arthritis, and one of a child with atlantoaxial subluxation, as the sole presenting features of JRA [11,12]. Therefore, chronic arthritis would be unlikely in a child with no other signs of joint inflammation, especially considering the rapid resolution of effusions while off anti-inflammatory medications.

Reactive arthritis affecting the cervical spine and TMJs has been reported in adults [13,14][15]. Associated organisms have included *Yersinia enterocolitica*, *Chlamydia trachomatis*, *Mycoplasma fermentans*, and *Mycoplasma genitalium*. This type of arthritis generally occurs 1–4 weeks following acute infection and often requires anti-inflammatory medications to subside. In our child, trismus and decreased neck extension occurred at the most acute phase of his infection, but they did follow his initial symptoms of neck pain and swelling by over one week. Although the child was pre-treated with antibiotics and microbacterial analysis of joint fluid was not available, the temporal association of his arthritis and aseptic meningitis is most consistent with a reactive arthritis. During his hospital course, the child's recurrent fevers were treated with ibuprofen and acetaminophen, and it is likely that the non-steroidal anti-inflammatory agent, ibuprofen, contributed to the improvement in his jaw and neck mobility. It is of note that the child was of Native American heritage, a population with a high HLA-B27 frequency and associated spondyloarthropathies [16]. Therefore, reactive arthritis should be considered in cases of transient TMJ and cervical spine effusions in the absence of findings suggestive of trauma, chronic arthritis, or septic joints.
